# A case report of ophthalmic artery emboli secondary to Calcium Hydroxylapatite filler injection for nose augmentation- long-term outcome

**DOI:** 10.1186/s12886-016-0276-3

**Published:** 2016-07-08

**Authors:** Eyal Cohen, Yossi Yatziv, Igal Leibovitch, Anat Kesler, Ran Ben Cnaan, Ainat Klein, Dafna Goldenberg, Zohar Habot-Wilner

**Affiliations:** Division of Ophthalmology, Tel Aviv Medical Center, Sackler Faculty of Medicine, Tel Aviv University, 6 Weizmann Street, Tel Aviv, 6423906 Israel

**Keywords:** Branch retinal artery occlusion, Calcium hydroxylapetite filler, Choroidal emboli, Ophthalmoplegia, Visual field

## Abstract

**Background:**

Filler injection for face augmentation is a common cosmetic procedure in the last decades, in our case report we describe long-term outcomes of a devastating complication of ophthalmic artery emboli following Calcium Hydroxylapatite filler injection to the nose bridge.

**Case presentation:**

A healthy 24-year-old women received a Calcium Hydroxylapatite filler injection to her nose bridge for the correction of nose asymmetry 8 years post rhinoplasty. She developed sudden right eye ocular pain and visual disturbances. Visual acuity was 20/20 in both eyes and visual field in the right eye showed inferior arch with fixation sparing and supero-temporal central scotoma. Examination revealed marked periorbital edema and hematoma, ptosis, ocular movements limitation, an infero-temporal branch retinal artery occlusion and multiple choroidal emboli. Eighteen months post initial presentation ptosis and eye movements returned normal and choroidal emboli absorbed almost completely. However, visual acuity declined to 20/60, visual field showed severe progressive deterioration with a central and supero-nasal field remnant and the optic disc became pallor.

**Conclusion:**

Cosmetic injection of calcium hydroxylapatite to the nose bridge can result in arterial emboli to the ophthalmic system with optic nerve, retinal and choroidal involvement causing long term severe visual acuity and visual field impairment.

## Background

Aesthetic soft-tissue filler injection for face augmentation has gained popularity in last decade, owing their relatively easy nonsurgical delivery, rapid results and low cost office based procedure. During the last years a growing amount of complications have been related to the procedure including allergic reaction, granuloma formation, skin necrosis or cellulitis [1] as well as ophthalmic and retinal artery occlusion or embolization [2–4]. Calcium hydroxylapetite (CaHA) is a semipermanent soft tissue filler, which may last 1 to 2 years in tissue. It is used mainly for face augmentation and is well adopted for the correction of post rhinoplasty deficiencies and asymmetries [5]. To date, only three case reports published on ocular embolism post CaHA injection to the glabella and nose bridge [6–8]. These cases had very poor initial visual acuities and only two cases had a short-term follow-up. We report a case of CaHA emboli to the choroid and retinal artery, demonstrated by spectral domain optical coherence tomography (SD-OCT), with a long-term follow-up. In our case, initial visual acuity was preserved but during 18 months follow-up visual acuity and visual field gradually deteriorated.

## Case presentation

A healthy 24-year-old female, underwent injection of CaHA to her nose bridge for the correction of nose asymmetry, 8 years post rhinoplasty. The injection was carried out by an ophthalmologist who is not an oculoplastic specialist. Immediately after the injection the patient complained of right eye (RE) periocular pain and blurred vision. Attempts were made to withdraw material from the injection site by aspiration, in addition to hot water compresses and topical massage.

**Fig. 1:**
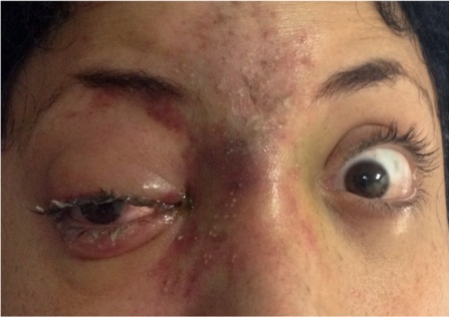
Facial photograph at presentation. Skin bruising on the nose bridge and forehead, ptosis and right eye exotropia

**Fig. 2:**
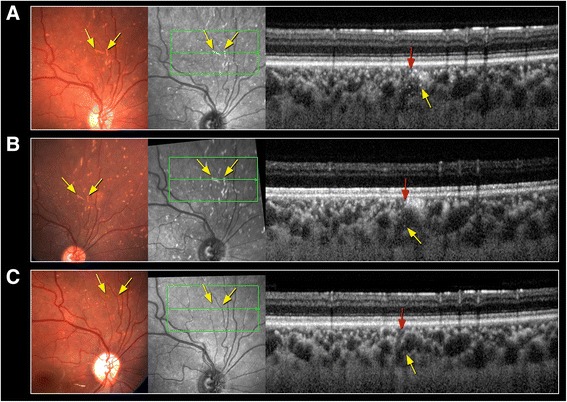
Fundus color picture, infrared and enhanced depth imaging SD-OCT - choroidal emboli. **a** At presentation: color picture and infrared - deep bright emboli (*yellow arrows*). SD-OCT - choriocapillary emboli (*red arrow*), and sattler’s layer choroidal emboli (*yellow arrows*). **b** Two months post presentation: partial absorption of emboli as shown in color picture, infrared and SD-OCT (*yellow and red arrows*). **c** Six months post presentation: additional absorption of emboli as shown in color picture and infrared (*yellow arrows*), SD-OCT - no emboli detected (*yellow and red arrows*)

**Fig. 3:**
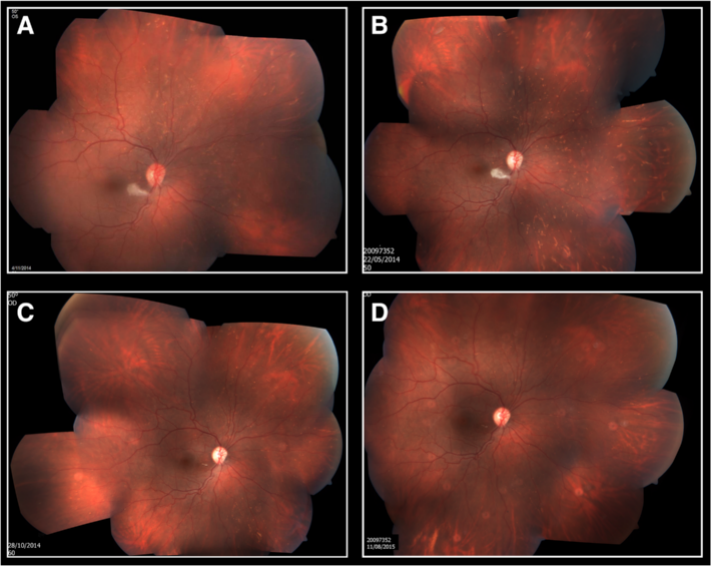
Right eye Fundus color picture. **a** At presentation: diffusely distributed choroidal emboli in superior, nasal and inferior quadrant. **b** Two months post presentation: partial absorption of emboli, optic disc pallor. **c** Six months post presentation: prominent absorption of emboli, optic disc pallor. **d** Eighteen months post presentation: choroidal emboli almost completely absorbed

**Fig. 4:**
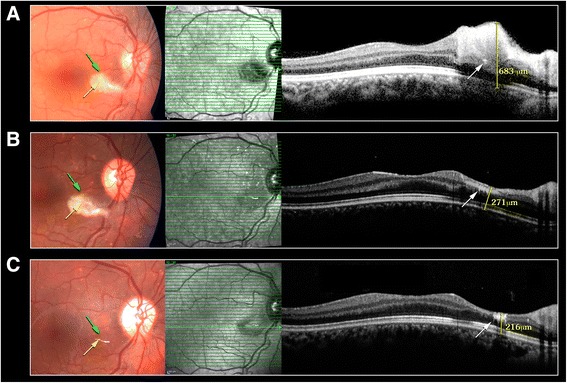
Fundus color picture, infrared and SD-OCT: color picture- CaHA emboli in the infero-temporal branch of the retinal artery (*yellow arrow*), CWS (*green arrow*). SD-OCT- intra-arterial emboli (*white arrows*), retinal thickness (*yellow caliper*). **a** At presentation. **b** Two months post presentation. **c** Six months post presentation

**Fig. 5:**
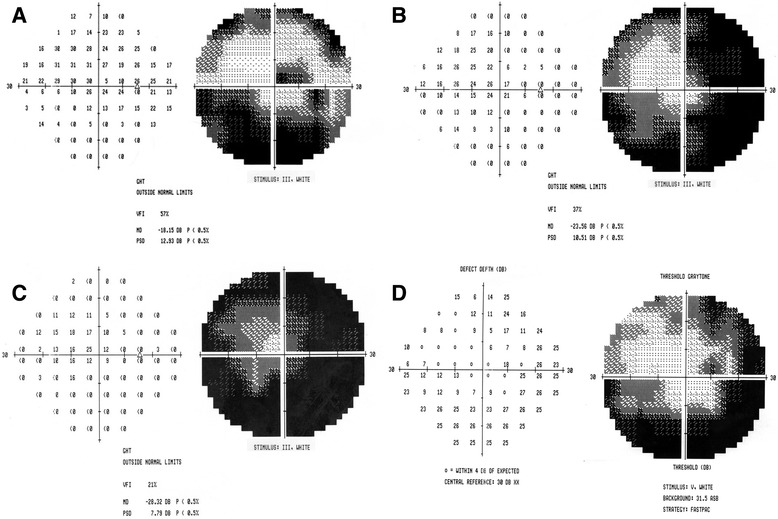
Right eye 30–2 Humphrey visual field examination. **a** At presentation: extensive inferior scotoma with fixation sparing, supero-temporal ceco-central scotoma. **b** Two months post presentation: marked deterioration showing inferior and temporal deep scotoma with central and nasal field sparing. **c** Six months post presentation: further deterioration showing a nasal field remnant. **d** Eighteen months post presentation: stimulus V showing inferior deep visual filed defect and marked peripheral superior decline in sensitivity

On examination 3 h post injection, visual acuity (VA) was 20/20 in both eyes. RE examination revealed no relative afferent pupillary defect (RAPD), a periorbital hematoma, skin bruising on the nose bridge and forehead, ptosis with mild levator function, prominent conjunctival congestion and RE exotropia (Fig. 1). In addition, limitation in adduction, superior and inferior eye movements were found. Fundus examination revealed deep yellowish emboli which appeared as choroidal emboli at the level of the choriocapillaris and sattler’s layer on SD-OCT enhanced depth imaging (EDI) (Fig. 2a). Emboli were scattered diffusely mostly in the superior, inferior and nasal parts of the choroid (Fig. 3a). CaHA emboli were also found in the infero-temporal branch of the retinal artery causing an occlusion with an adjacent cotton-wool spot (Fig. 4a). Choroidal thickness was normal and equal in both eyes. RE 30–2 Humphrey visual fields (VF) showed extensive inferior scotoma with fixation sparing which can be explained by the massive distribution of emboli in the superior retina, supero-temporal ceco-central scotoma corresponding to the area of the infero-temporal branch artery occlusion and superior peripheral scotoma corresponding to the upper eyelid swelling and ptosis (Fig. 5a). The patient refused fluorescein and indocyanine green angiography examinations. Left eye (LE) examination was normal. Head and orbits computed tomography (CT) demonstrated linear deposits with bone density in the right medial upper eyelid and nose bridge, CT angiography showed no arterial occlusion and on CT venography there were no sinus vein filling defects. No signs of central nervous system infarction where demonstrated. Initial treatment included Enoxaparin sodium (Clexane, Aventis, France) 60 mg twice a day for 2 days, which was discontinued after exclusion of thrombotic event. Acetylsalicylic acid (Micropirin, Dexcel pharma, Israel) 100 mg per day, Amoxicillin/Clavulanate (Augmentin, GlaxoSmithKline, UK) 875 mg twice a day for 7 days and Prednisone (Prednisone, Rekah, Israel) 60 mg per day which was gradually tapered down. Topical antibiotics – Ofloxacin 0.3 % (Oflox, ALLERGAN, Ireland) eye drops and Mupirocin (Bactroban, GlaxoSmithKline, UK) ointment to the nose bridge and forehead area were given for 14 days. Two months post initial treatment, best corrected visual acuity (BCVA) was 20/32 in the RE and 20/20 in the LE. RE examination revealed normal eyelids, normal eye movements, with no RAPD. The optic disc became pale, the cotton wool spot along the infero-temporal branch of the retinal artery partially absorbed (Fig. 4b) but the CaHA emboli were still seen in the fundus photograph (Fig. 3b). The EDI-OCT reveal some degree of absorption of the CaHA emboli in the choroid as the hyper reflective choroidal dots became less prominent (Fig. 2b). 30–2 Humphrey VF revealed marked deterioration; inferior and temporal deep scotoma with central and nasal field sparing (Fig. 5b). Six months post treatment initiation BCVA was 20/40 in the RE and 20/20 in the LE. The infero-temporal artery was occluded by CaHA emboli (Fig. 4c) and the amount of the choroidal emboli was significantly reduced (Figs. 2c and 3c). Visual field examination further deteriorated, showing a central nasal field remnant (Fig. 5c). At the end of follow up 18 months post initial presentation BCVA was 20/60 in the RE, choroidal emboli almost completely absorbed (Fig. 3d). Visual field examination further deteriorated, stimulus III can not be performed and stimulus V showing inferior deep visual filed defect and marked peripheral superior decline in sensitivity (Fig. 5d)

## Discussion

Our case demonstrates long-term results of a devastating ocular complication post CaHA filler injection to the nose bridge. CaHA emboli to the arterial ophthalmic system caused partial ophthalmoplegia, branch retinal artery occlusion, and multiple choroidal emboli. Vascular related events are major complications of soft tissue filler injection and can occur from direct needle injury to the vessels, external compression of vessels by surrounding filler or intravascular embolism of injected material. Intra-arterial embolism formation after soft tissue filler injection to the nose bridge may be caused by anterograde arterial flow of material injected trough artery-vein anastomosis found in the nose mucosa, or by retrograde arterial displacement of the injected product. The latter can occur when injection pressure is higher than systolic arterial pressure and the injected product moves from peripheral arterial vessels into proximal arterial vessels. After stopping the injection the product flow with the blood stream to peripheral arterial branches and cause an embolic event [2–4]. We assume that in our case the CaHA was directly injected to the dorsal nasal artery which is a peripheral artery in the ophthalmic system and then moved retrograde with the blood stream to the various branches of the ophthalmic artery. Involvement of the central retinal artery and posterior ciliary artery may explain the branch retinal artery and multiple choroidal emboli. Supraorbital, infraorbital and muscular arteries emboli may explain the ophthalmoplegia and ptosis.

To date, only 3 cases on ocular complications following CaHA filler injection were published [6–8]. Of them, only two cases reported on posterior segment involvement. The first case was published by Kim et al. [6] and demonstrated bilateral blindness after CaHA filler injection for nose augmentation. The patient presented with local skin necrosis, bilateral total ophthalmoplegia, anterior chamber ischemia and ophthalmic artery obstruction with multiple retinal arteries and choroidal emboli. Initial VA was no light perception in both eyes. Unfortunately no information regarding treatment or follow up was provided. Hsiao et al. [7] recently published a case of unilateral distal retinal arteries and choroidal emboli post CaHA injection to the glabella. Initial VA was hand motion. Management included various topical and systemic treatments, at 3 month follow up VA achieved 20/200.

Our case is the first to demonstrate CaHA emboli documented over 18 months follow up. In our case, the patient initially presented with preserved VA. Treatment included anticoagulants and systemic corticosteroids as recommended in cases suspected of intravascular filler injection [9]. At 18 months follow-up, VA and VF markedly deteriorated although choroidal emboli were reduced significantly. We assume that massive amount of CaHA emboli to the ophthalmic artery branches caused a significant decrease in retinal, choroidal and optic nerve perfusion resulting in irreversible VF damage with progressive deterioration of VF and VA over time. Ptosis and ophthalmoplegia completely resolved which may be explained by renewal of arterial flow and muscle cells rehabilitation.

## Conclusion

Ophthalmic artery embolization secondary to CaHA filler injection to the nose bridge may cause a devastating long-term outcome. Physicians should be aware of this complication and inform their patients before offering these cosmetic treatments.

## Abbreviations

BCVA, best corrected visual acuity; CaHA, calcium hydroxylapetite; EDI, enhanced depth imaging; LE, left eye; RAPD, relative afferent pupillary defect; RE, right eye; SD-OCT, spectral domain optical coherence tomography; VA, visual acuity; VF, visual fields
